# Contextual Features of the Cue Enter Episodic Bindings in Task Switching

**DOI:** 10.5334/joc.220

**Published:** 2022-04-18

**Authors:** Elena Benini, Iring Koch, Susanne Mayr, Christian Frings, Andrea M. Philipp

**Affiliations:** 1 Chair of Cognitive and Experimental Psychology, Institute of Psychology, RWTH Aachen University, Germany; 2 Chair of Psychology and Human-Machine Interaction, University of Passau, Germany; 3 Chair of General Psychology and Methodology, Department of Cognitive Psychology, University of Trier, Germany

**Keywords:** binding, context, stimulus modality, stimulus language, response-repetition effect

## Abstract

Evidence suggests that the features of a stimulus and the actions performed on it are bound together into a coherent mental representation of the episode, which is retrieved from memory upon reencountering at least one of these features. Effects of such binding and retrieval processes emerge in action control, such as in multitasking situations like task switching. In the task-switching paradigm, response-repetition benefits are observed in task repetitions, but response-repetition costs in task switches. This interaction of task repetition (vs. switch) with response repetition (vs. switch) may be explained in terms of task-response binding. In two experiments, we included a task-irrelevant contextual feature in a cued task-switching paradigm using word identification tasks. In Experiment 1, the cue modality could vary between visual and auditory; in Experiment 2, the cue language could vary between English and Spanish, while the target stimulus was always presented visually and in German. We predicted that repeating the contextual feature in the subsequent trial would retrieve the features of the previous trial, even though cue modality or cue language did not afford any response and were not associated with either task. The results showed that response repetition-benefits in task repetitions were observable when the context (i.e., the modality or the language of the cue) repeated but disappeared when the context switched from the previous trial. These results are consistent with context-specific binding and retrieval processes in task switching.

Humans need to interact with and respond to a continuous flow of stimuli and not all of them are relevant for their scopes. Associative mechanisms that routinize the response to certain reoccurring stimuli facilitate the interaction with such a complex environment. Sometimes, however, also irrelevant stimuli become associated with behavioural responses (Frings et al., 2007; Mayr & Buchner, 2006; Rubin & Koch, 2006). In the present study, we explore how irrelevant stimulus features are associated, or bound, with task-relevant features and the executed response and how this impacts humans’ task-switching performance. Specifically, using cued task switching, we tested whether cue modality features (i.e., visual vs. auditory presentation of the cue) and cue language features (i.e., Spanish vs. English cue language) are bound with the task and the response.

Binding and retrieval mechanisms may be the building blocks of associative learning processes (Moeller & Frings, 2017; Schmidt et al., 2020) and indicate the automatic association, or binding, of the perceptual features of a stimulus and the actions performed on it (i.e., the response). Different accounts like the Theory of Event Coding (TEC; Hommel, 1998, 2004, 2019) or the Binding and Retrieval in Action Control framework (BRAC; Frings et al., 2020) maintain that such associations are stored in memory as a coherent representation of the recent episode and are retrieved from memory whenever at least one feature, or response, re-occurs. Therefore, binding and retrieval processes can automatize responses to certain stimuli, making the interaction with the environment more efficient (Logan, 1988). However, binding and retrieval processes may as well result in performance costs, for example when the same response has to be performed on a different stimulus.

## Feature binding in task switching

In the present study, we were mainly interested in binding and retrieval effects of irrelevant contextual features in cued task switching (cf. Benini et al., 2022; Kandalowski et al., 2020; Koch et al., 2018; Rubin & Koch, 2006). In task switching, participants switch between two or more tasks on a trial-to-trial basis (see Kiesel et al., 2010; Koch, Poljac et al., 2018; Monsell, 2003; Vandierendonck et al., 2010, for reviews on task switching). When the task sequence is unpredictable, the task to perform in each trial is signalled by a cue.

In task switching, repeating the same task as in the previous trial results in a faster and more accurate response than switching the task. Furthermore, repeating the *response* from the previous trial typically results in a performance benefit in task repetitions, but it turns into a cost in task switches, the so-called response-repetition (RR) effect (Altmann, 2011; Druey, 2014; Grzyb & Hübner, 2013; Kiesel et al., 2010; Kleinsorge, 1999; Koch et al., 2011; Rogers & Monsell, 1995; Schuch & Koch, 2004, 2010). This RR effect could be due to the binding between the task and the response: Repeating a task retrieves the previous response, which is incorrect in response switches (Altmann, 2011; Schuch & Koch, 2004, but see Hübner & Druey, 2006). In contrast, in task switches, the binding of the task and the response leads to a partial mismatch in response repetitions, as the same response is given to a different task. Such a binding account assumes that the task and the response are bound in each trial. Then, retrieval of the previous response may or may not take place when the task repeats versus switches, respectively. The BRAC framework (Frings et al., 2020) emphasizes the independence between binding and retrieval processes. Thus, it constitutes a suitable framework to describe RR effects in task switching.

A prominent alternative explanation of the RR effect assumes that the just-executed response is inhibited (Druey, 2014; Hübner & Druey, 2006; see Gade et al., 2014, for a review) and this causes RR costs in task switches. However, the inhibition is overcome in task repetitions as the repeating stimulus category primes the previous response causing RR benefits.

## Binding of irrelevant features in task switching

Three previous studies (Benini et al., 2022; Kandalowski et al., 2020; Koch et al., 2018) explored the influence of a contextual, task-irrelevant feature (the *context* henceforth) on the RR effect in task switching.

Kandalowski et al. (2020) and Koch et al. (2018) asked their participants to switch between a magnitude and a parity task. The authors included cue modality as the context. That is, in each trial, the cue could be a black bar (visual modality) or a tone (auditory modality). The black bar could be presented at the top versus the bottom of the screen; the tone could be high- versus low-pitched. The two “high” cues (i.e., the bar at the top of the screen and the high-pitched tone) were always assigned to the same task, for example, the magnitude task; the two “low” cues to the other task, for example, the parity task. Koch et al. (2018) showed that repeating the response in task repetitions, compared to switching it, yielded a larger performance benefit in context repetitions (i.e., cue modality repetitions) than switches. A similar data pattern was observed in Experiment 2 by Kandalowski et al. (2020), albeit nonsignificant. Benini et al. (2022) replicated this pattern by again observing that the RR benefits in task repetitions increased when the context repeated compared to when it switched.

Such modulation of the RR benefit by the context is consistent with the assumption that the context, despite being task-irrelevant, is bound with the task and the response. Then, when the context repeats in the next trial, it retrieves the previous task and response.

In contrast, the RR costs in task switches were never modulated by context repetitions versus switches in the three studies. The presence of context modulation in task repetitions and its absence in task switches may result from the sum of feature binding and response inhibition processes (see Koch et al., 2018). Hence, binding effects may coexist with response inhibition.

Another explanation for the absence of context modulation on RR costs in task switches might involve the hierarchy of the features. The task may be represented on a hierarchically higher level than the response and the context (Kleinsorge & Heuer, 1999; von Bastian & Druey, 2017; see Hommel, 2004, 2019 for the concept of feature weighting). Hence, the context may only influence response repetitions when the task is repeated. Similarly, a task switch may increase the discriminability of the current and previous events (see Whitehead, Pfeuffer & Egner, 2020, for a similar discussion). That is, switching the task may prevent any context-triggered retrieval by rendering the current trial clearly distinct from the previous.

Different studies instead found that context repetitions versus switches modulated the RR costs in task switches. Dreisbach and Wenke (2011) and Dreisbach and Haider (2008) introduced the colour or the font of the target as an irrelevant context. They found that repeating versus switching the context did not modulate RR effects in task repetitions nor in a single-task condition (in which all trials are task repetitions). That is, repeating the irrelevant context did not result in a specific benefit of repeating the response as well. On the contrary, RR costs in task switches were larger when context repeated and reversed (i.e., into benefits) when context switched.

We can speculate that Koch et al. (2018) and Benini et al. (2022) could better divide cue- from target-triggered processes by presenting the cue before the target (200 ms or 600 ms, respectively). In contrast, Dreisbach and colleagues used no cue. Hence, participants retrieved the correct task and selected the response and encoded the target and saw the context feature all at the same time. The temporal overlap of such processes could reduce the impact of repeating the context, by reducing the strength of the retrieval (see Benini et al., 2022 for a discussion).

## Cue encoding and context-triggered retrieval

In Benini et al. (2022), Kandalowski et al. (2020) and Koch et al. (2018), the cue played two major roles. On the one hand, the cue indicated and retrieved the relevant task in each trial (Arrington et al., 2007; Gade & Steinhauser, 2020; Monsell & Mizon, 2006); on the other hand, it set the context feature.

In these studies, repeating the context in a task repetition also implied repeating the cue. Hence, repeating the context in a task repetition may prime cue encoding compared to switching the context (e.g., Logan & Bundesen, 2003). Such cue repetition benefits may contribute to the RR benefits in task repetitions and their reduction when the context switched (i.e., when the task repeated, but the cue was different).

Such cue repetition benefits were largely investigated in task switching by studies using two (or more) cues for each task, that is, using a 2:1 cue:task mapping (e.g., Forrest et al., 2014; Logan & Bundesen, 2003; Monsell & Mizon, 2006). However, such a 2:1 cue:task mapping does not allow context repetition in task switches, in contrast with Benini et al. (2022), Kandalowski et al. (2020), Koch et al. (2018) and the present study. In other words, the latter studies partially resemble 2:1 cue:task mapping studies, as they allow cue switches in task repetitions. However, they introduce an important novelty, which is the possibility to observe context repetitions in task switches. Furthermore, they are inserted in a different theoretical framework, aimed at measuring context binding and retrieval effects.

## The present study

In the present study, we first replicated Kandalowski et al. (2020) and Koch et al. (2018), using cue modality (visual vs. auditory) as the context feature. However, we used different, verbal materials. Then, we used the same verbal materials in Experiment 2, where the context was cue language (English vs. Spanish). We adopted a feature binding approach to account for the effect of switching versus repeating the context (Frings et al., 2020). That is, we investigated whether a repeating context would retrieve the previous task and response. Specifically, we examined whether such a mechanism extends to abstract context features, which were not examined before. Generalizing such results is important given the assumed ubiquity of binding and retrieval processes in action control (Frings et al., 2020; Henson et al., 2014).

## Experiment 1

In Experiment 1, each task was cued by a word that was presented in either the auditory or the visual modality. That is, participants could either read or hear the word “Leben” (“life” in German for the living/non-living categorization) or the word “Format” (“shape” in German for the bigger/smaller categorization).

Using words as the cues also implied that cue processing relied on word recognition, differently from Benini et al. (2022; Experiment 1), Kandalowski et al. (2020) and Koch et al. (2018). We used visually presented German words as target stimuli, thereby converting also target processing into a word recognition process. German was the participants’ native language.

According to the Logogen model by Morton (1979, 1980), word recognition proceeds through different pathways in the auditory and visual modalities. Therefore, in Experiment 1, participants switched between two processing pathways based on the cue modality, which switched or repeated unpredictably. Previous research has established that a modality switch impairs performance compared to a repetition (Lukas et al., 2010a, 2010b, 2014; Spence & Driver, 1997), arguably due to the need to switch between processing pathways. Therefore, we expect performance to generally worsen in context switches compared to repetitions.

Note that when the cue was presented auditorily, participants also switched the modality *within* a trial, since the target word was always presented visually. The issue of such a difference between trials was addressed in Experiment 2.

Furthermore, we looked at the effect of switching versus repeating the irrelevant context feature from the previous trial in relation to switching versus repeating the relevant features. That is, we expected context switch versus repetition (i.e., *context relation*) to interact with task relation and response relation. Specifically, we predicted repeating the context to be beneficial when also the task and the response repeated, and detrimental when these both switched. Such a pattern would be consistent with a binding perspective which maintains that all the features of a trial are bound together and that such bindings can be retrieved upon repetition of one or more features.

### Materials and methods

#### Participants

We managed to collect 48 participants. In previous similar studies, the effect size of the three-way interaction of task relation, response relation, and context relation ranged from small (η_p_^2^ = 0.03; Benini et al., 2022; Experiment 1) to large (η_p_^2^ = 0.58; Koch et al., 2018; Experiment 1B). With 48 participants, considering the interaction effects as difference scores, we achieved a power of .80 to detect a small-to-medium effect size of dz = 0.41 (two-tailed paired-sample t-test; as calculated with G*, Erdfelder et al., 2009; Faul et al., 2007).

Four students from the RWTH University were recruited via email and took part in exchange for partial course credits. The remaining 44 participants were recruited via Prolific (*https://www.prolific.co/*) and were pre-screened according to their age (between 18 and 35 years), native language (German) and their English language ability (fluent). They were rewarded with £5.63 for an estimated completion time of 45 minutes (£7.5/h). We stopped the data collection after reaching 44 submissions with an average error rate below 20%.

Our final sample included 48 participants (16 females, mean age = 25.3 (±5.4), four did not declare their age), with an average error rate equal to 6.6%. Nine were left-handed.

The procedure of this experiment, as well as Experiment 2, was approved by the ethics committee of the Philosophical Faculty at RWTH Aachen University. Neither of the two experiments was preregistered. The data and the analyses scripts are available at: *http://dx.doi.org/10.23668/psycharchives.5240*, whereas the experimental materials are available from the corresponding author on request.

#### Stimuli, tasks, and responses

Target stimuli consisted of 24 German words (see ***Table 1***) representing objects or animals that participants categorized based on two possible tasks. One task required deciding whether the object/animal was bigger or smaller than a typical shoebox and was cued by the word ‘FORMAT’ (i.e., “shape”). The other task required deciding whether the target was living versus non-living and was cued by the word ‘LEBEN’ (i.e., “life”). The cue words were presented either visually, written in blue ink, at the midway between the centre and the top of the screen, or auditorily, via earphones or headphones. All target words were presented visually at the centre of the screen and written in black. All the visually presented words were displayed on a white background in Arial font 40 and occupied 37 pixels vertically. Horizontally, they occupied between 103 and 197 pixels.^1^ Participants were instructed to respond by pressing the A or the L key on their keyboard using their left and right index fingers respectively and to lay the fingers on the keys for the whole duration of the experiment. The response-key mapping was counterbalanced across participants.

**Table 1:** Target stimuli and the correct responses to the two tasks.


WORD	LIFE/VIDA/LEBEN	SHAPE/FORMA/FORMAT

KAEFER	living	smaller

VOGEL	living	smaller

KNOPF	non-living	smaller

AUTO	non-living	bigger

ERBSE	living	smaller

SCHRANK	non-living	bigger

BLATT	living	smaller

TASSE	non-living	smaller

HIRSCH	living	bigger

ZAUN	non-living	bigger

BLUME	living	smaller

GABEL	non-living	smaller

BRILLE	non-living	smaller

PFERD	living	bigger

AFFE	living	bigger

ZWIEBEL	living	smaller

TELLER	non-living	smaller

DUSCHE	non-living	bigger

BAUM	living	bigger

FENSTER	non-living	bigger

FRAU	living	bigger

STUHL	non-living	bigger

MESSER	non-living	smaller

ZIEGE	living	bigger


#### Procedure

The experiment was programmed in, and hosted by, Gorilla Experiment Builder (Anwyl-Irvine et al., 2020). Participants could only take part using a desktop computer or a laptop and Chrome as the browser. RWTH students were sent an email with the link to the experiment, while the remaining participants accessed the experiment through their Prolific account. In both cases, they could start at any time and had a maximum of one hour and 30 minutes to complete the experiment, against an average completion time of 36 minutes.

Upon access, participants were asked to pick a quiet place and to assume a comfortable position, to open the study in a new window and/or to enter full-screen mode pressing the F11 key. They read and accepted the informed consent and the data protection regulation. After that, they were required to plug in non-Bluetooth earphones or headphones. To ensure that participants were wearing headphones or earphones, we implemented a screening task suggested by Woods et al. (2017) which consists of discriminating which of three tones is the softest. This task becomes extremely difficult if performed without headphones or earphones. A further screening verified that the participants’ browsers could play audio files and, finally, we ensured that participants knew the German words we used as stimuli. To do so, they were presented with 24 images, one for each target word, which they had to name by selecting the correct word among 7 other distractors, which were randomly drawn from the set of target words. The participants could only proceed to the following image after selecting the correct response. They were allowed to continue with the experiment only if they committed less than 4 errors in total. Participants who were excluded from the experiment at any of these preliminary stages were compensated with the same hourly wage, based on the time they spent in the experiment. Five participants voluntary dropped out of the experiment after having given their consent. Thus, we did not collect any data from them.

Finally, participants started the main task by reading the instructions. These were presented in English and mentioned that the cue modality (visual vs. auditory) was not relevant for their tasks.

The participants completed 24 training trials identical to the experimental trials, except for the presence of accuracy and speed feedback in the training trials. The sentence: “Please, try to be faster!” was presented for 400 ms if no response was given after 2000 ms, and a red cross would appear for 200 ms following an incorrect response, while a green tick indicated a correct response. Upon completing the training, they could decide to read the instructions again and to repeat the training.

Each trial started with a fixation cross, displayed for 1400 ms. The cue was presented right after for 1000 ms, after which it disappeared if it was presented visually. The duration was chosen to match the duration of the auditory cue utterance so that the cue-target interval was constant across visual and auditory cues. The target appeared as soon as cue presentation ended, and it remained on the screen for a maximum of 2500 ms or until a response was given. The new trial started right away with the fixation cross.

The experiment consisted of 4 blocks of 96 trials each. Between the blocks, participants were shown their overall accuracy and were reminded of the key-response mapping. Before starting a new block, participants were briefly reminded to place their index fingers on the A and L keys and not to move them.

Each target word was presented twice for each task in each block, once cued by a visual and once by an auditory cue. No direct target repetitions were allowed and the difference between the number of task switches and repetitions was equal to either 1 or – 1. We did not constrain the number of correct response and context repetitions, but these were balanced in the data we analysed (repetitions were 48.7% and 51.6%, respectively). Targets and tasks sequence was pseudorandomized according to these constraints so that participants could not predict the upcoming cue modality, task or target. After completing the 4 blocks, participants were thanked and debriefed through a written text explaining the rationale of the experiment.

#### Design

Our experiment had a 2 × 2 × 2 within-subjects design. To test our hypotheses, we ran two ANOVAs with reaction times or error rates as the dependent variables, and task relation (repetition vs. switch), response relation (repetition vs. switch), and context relation (repetition vs. switch) as the within-subjects independent variables. As we were mostiy interested to see whether a response-repetition benefit in task repetitions was modulated by context repetitions versus switches, we further calculated paired-sample t-tests comparing response repetition and response switch trials in these specific conditions.

### Results

#### Reaction times

To analyse participants’ reaction times (RTs), we excluded trials slower than three standard deviations from each participant’s mean. Furthermore, we excluded error and post-error trials, trials faster than 200 ms, and the first trial of each block. We, therefore, removed 14.2% of the raw data.

The ANOVA revealed a significant main effect of task relation, *F*(1, 47) = 38.27, *p* < 0.001, η_p_^2^ = 0.449, indicating task repetition benefits (795 ms vs. 843 ms, for task repetitions vs. switches, respectively). Task relation also entered in a significant two-way interaction with response relation, *F*(1, 47) = 34.11, *p* < 0.001, η_p_^2^ = 0.421, such that the RR benefits in task repetitions (33 ms) turned into costs in task switches (–30 ms), replicating the standard two-way interaction (see, for example, Altmann, 2011; Schuch & Koch, 2004).

The main effect of context relation was significant, *F*(1, 47) = 29.93, *p* < 0.001, η_p_^2^ = 0.389, indicating context-repetition benefits (803 ms vs. 836 ms, for context repetitions vs. switches, respectively). Context relation also entered in a significant two-way interaction with response relation, *F*(1, 47) = 5.47, *p* = 0.024, η_p_^2^ = 0.104, such that the small RR benefits in context repetitions (7 ms) turned into costs in context switch trials (–15 ms).

No other main effect or interaction was significant (all *F*s < 0.86, all *p*s > 0.357).

Although the three-way interaction of task relation, response relation and context relation was not significant, we ran two exploratory paired-sample t-tests to examine the RR benefits in task repetitions in context repetitions versus context switches. The RR benefit was significant in context repetitions (35 ms, *t*(1,47) = 3.7, *p* < 0.001, *d_z_* = 0.53), but not in context switches (9 ms, *t*(1,47) = 1.11, *p* = 0.273, *d_z_* = 0.16. The Bonferroni-adjusted alpha was equal to .025).

#### Error rates

To analyse participants’ error rates, we applied the same data cleaning as for the reaction times, but we kept the error trials. This led to removing 8.9% of the observations.

The ANOVA revealed a main effect of task relation, *F*(1, 47) = 23.69, *p* < 0.001, η_p_^2^ = 0.335, indicating task repetition benefits (4.9% vs. 7.2%), and a significant interaction between task relation and response relation, *F*(1, 47) = 9.62, *p* = 0.003, η_p_^2^ = 0.170, such that the small RR benefits in task repetitions (0.63%) turned into costs in task switches (–1.84%).

We found a significant main effect of context relation, *F*(1, 47) = 5.25, *p* = 0.026, η_p_^2^ = 0.101, indicating general context repetition *costs* (–6.4% vs. –5.7%), opposed to the context-repetition benefits in the RTs. Context relation also entered in a significant two-way interaction with response relation, *F*(1, 47) = 11.10, *p* = 0.002, η_p_^2^ = 0.191, so that, as in the RTs data, the small RR benefits in context repetitions (0.36%) became costs in context switches (–1.57%).

As in the RTs, we explored the RR benefits in task repetitions in context repetitions versus switches. The RR benefits in task repetitions were neither significant in context repetitions (1.02%, *t*(1,47) = 1.86, *p* = 0.069, *d_z_* = 0.27), nor in context switches (0.02%, *t*(1,47) = 0.31, *p* = 0.755, *d_z_* = 0.04. The Bonferroni-adjusted alpha was equal to .025).

### Discussion

**Figure 1:**
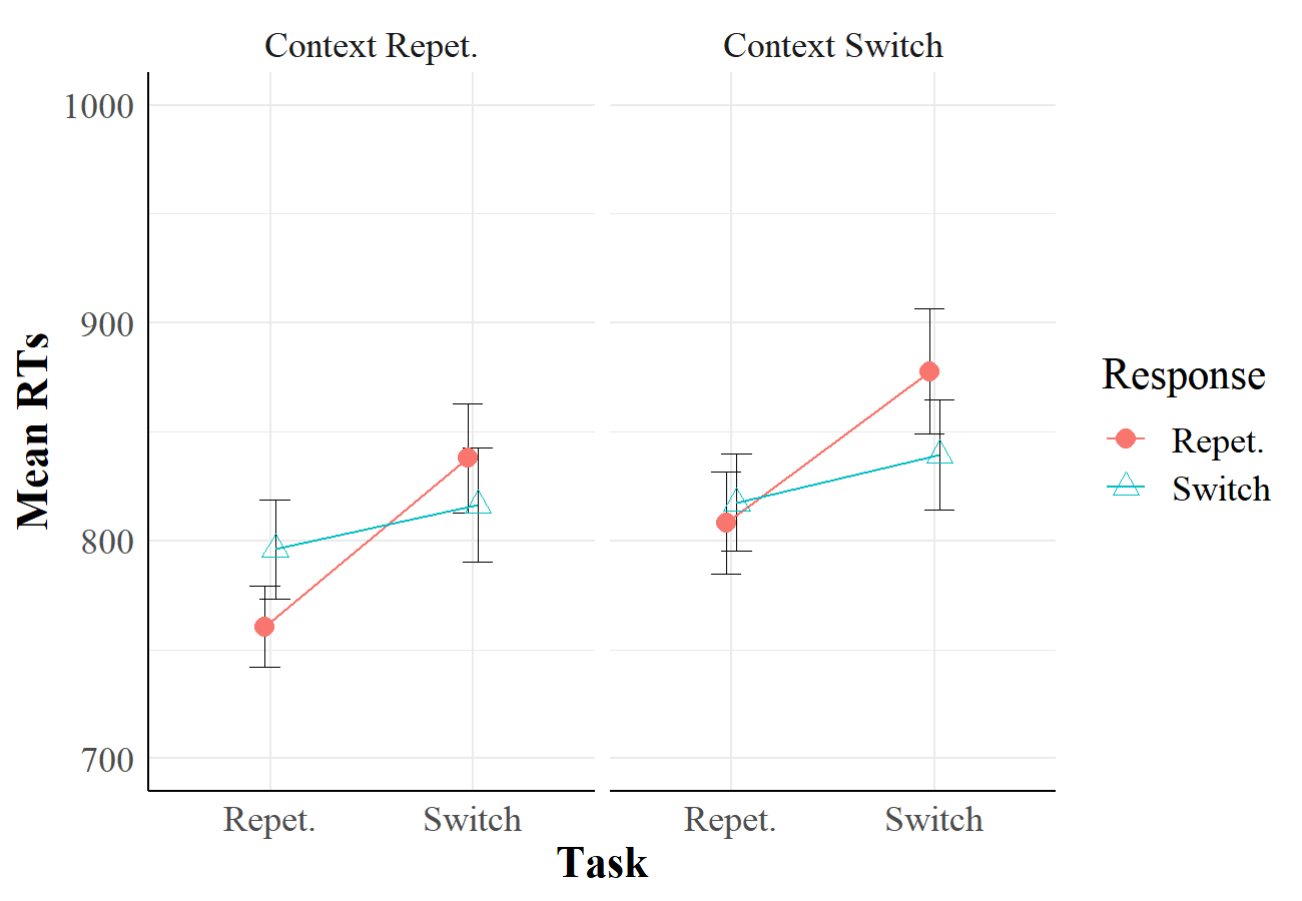
Mean RTs in Experiment 1. *Note*: Vertical bars represent standard errors of the mean.

**Figure 2:**
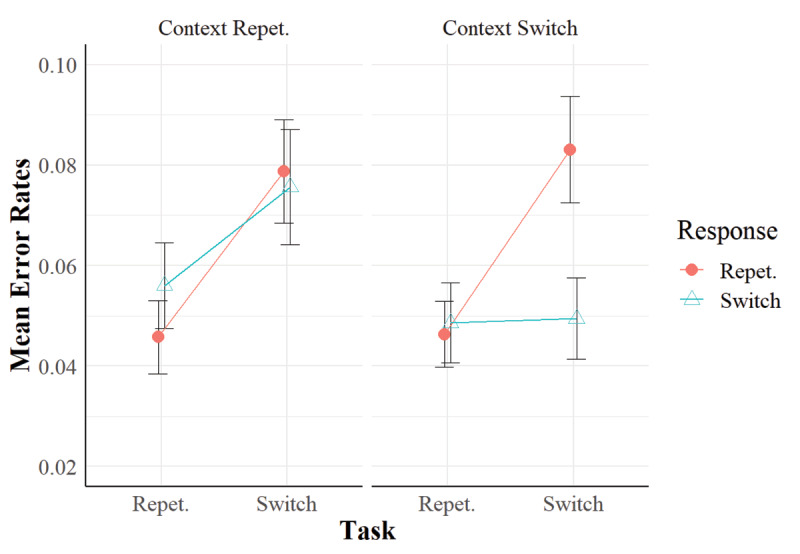
Mean Error Rates in Experiment 1. *Note*: Vertical bars represent standard errors of the mean.

The analyses showed that we replicated the task repetition benefits and the RR effect in our sample in both participants’ reaction times (***Figure 1***) and error rates (***Figure 2***). Importantly, they also showed that when the context repeated, despite being task-irrelevant, this implied a performance benefit when the response repeated. This result replicated across reaction times and error rates (see ***Figures 1*** and ***2***), despite the main effect of context went in opposite directions in the RTs (context repetition benefits) and the error rates (context repetition *costs*). However, the three-way interaction of task relation, response relation and context relation was not significant. Nonetheless, in the RTs, we found significant RR benefits in task repetitions when the context repeated, but not when it switched. This indicates a numerical trend in the data that suggests that the context was bound with the task and the response.

## Experiment 2

In Experiment 2, we used the same tasks and target stimuli as in Experiment 1, but the manipulated context feature was the language of the cue (either English or Spanish). We selected Spanish as it granted a large pool of participants in Prolific as compared to other European languages (e.g., French or Italian). Therefore, in Experiment 2, each task was cued by the same concept as in Experiment 1 (concepts of “shape” and “life”), but this concept was either conveyed via a Spanish or an English translation-equivalent lemma (i.e., the mental representation of a word).

Therefore, in each trial, participants needed to activate the relevant language lexicon and inhibit the potentially competing lexicons (see Declerck & Philipp, 2015 for a review on language switching and the mechanisms underlying multilingual language control), while stimulus modality was always visual.

Using the language as the context granted easy access to three different levels of the context (Spanish, English, or German). Therefore, in all trials, there was always a context switch *within* each trial in Experiment 2 (i.e., language transition between cue and target was either Spanish to German or English to German), as opposed to Experiment 1, in which a within-trial switch (from an auditory cue to a visual target) only took place in half of the trials. The other main difference between Experiment 1 and 2 consisted in the *level* at which the context switched in the two experiments. While in Experiment 1, as well as in Benini et al. (2022), Kandalowski et al. (2020) and Koch et al. (2018), a change in the context implied a change in the cue at the perceptual, surface level (cue modality or cue colour), in Experiment 2, the context changed at a conceptual, linguistic level. From a perceptual point of view instead, the cue words were quite similar across the two languages (black words of similar length printed on a white background).

Based on previous research (Declerck & Philipp, 2015), we expected a better performance in context repetition than in context switch trials. Moreover, we expected language (i.e., context) relation to interact with task relation and response relation, signalling that the RR effect is modulated by a repeating versus switching context. Our predictions are, therefore, the same as for Experiment 1, although a modality switch (i.e., as in Experiment 1) implies a change at the perceptual and not at the lexical level of the stimulus, while the opposite holds for a switch in the language of a written word (i.e., as in Experiment 2). That is, we expect context binding and retrieval effects to generalize beyond previous specific operationalizations of context. However, we note that the different types of contexts implied further differences in the two experiments (e.g., the within-trial switch and the switch between pathways). Thus, any difference in the binding effects in the two experiments will be also discussed in relation to the different processes elicited by stimulus modality versus language switching.

### Materials and methods

#### Participants

We collected data via Prolific (*https://www.prolific.co/*), pre-filtering the participants according to their age (between 18 and 35 years), their native language (German), and the number of languages they spoke (3 languages): We searched for German native speakers, fluent in both Spanish and English and we managed to collect 44 participants with an average error rate below 20% and with at least a basic knowledge of Spanish. We then removed a further participant whose average error rate was also greater than 20%, which we did not notice before. This left us with 43 participants (30 females), whose mean age was 26 ± 3 years old. Considering the interaction effects as difference scores, with 43 participants we had a probability of 80% to detect a small-medium effect size (*d_z_* = 0.42) with a two-tailed paired-sample t-test, as calculated with G*Power (Erdfelder et al., 2009; Faul et al., 2007).

Participants were rewarded with £4.38 against an estimated completion time of 35 minutes (£7.50/hour). Participants could only use a computer or laptop and Safari or Chrome as browsers.

#### Stimuli, tasks, and responses

The target stimuli and the tasks were the same as in Experiment 1. The cue words, however, were “FORMA” (shape) and “SHAPE” for the bigger/smaller categorization, and “VIDA” (life) and “LIFE” for the living/non-living categorization. To force participants to read the words and to avoid that they could rely on the length of the letter strings, we masked the cue words in short sequences of Zs so that the printed words had comparable length. Therefore, the four cues were: ZZFORMAZZ, ZZSHAPEZZ, ZZZVIDAZZZ, ZZZLIFEZZZ.^2^ This way, the maximum difference between cue-words lengths was 12 pixels (the shortest was LIFE which was 228 pixels long, and the longest was FORMA which was 240 pixels long). This also rendered the words more similar. All cue and target words were displayed on a white background in Arial font 40. Cue words were printed in blue, midway between the top and the centre of the screen. The target words were the same German words as in Experiment 1 and were displayed in the same way as in Experiment 1. Participants responded by pressing the A or the L key on their keyboard, which they were instructed to press with their left and right index finger respectively.

#### Procedure

The procedure was identical to Experiment 1, except for the tasks ensuring the pre-requisites for presenting auditory stimuli, which were substituted by a short Spanish test. It consisted in picking the correct English translation of a Spanish word, presented among eight options. In total, they translated 5 words, among which there were the two Spanish cue words: “VIDA” and “FORMA”. After that, they answered the following question: “Is your level of Spanish intermediate (A2-B1) or higher?”. Participants could continue with the experiment if they correctly translated at least three words *and* if they answered affirmatively to the question.^3^ Ten participants voluntary dropped out of the experiment before completing the main task.

Differently from Experiment 1, the cue word remained on the screen with the target word until the response or the following fixation cross.

In the data we analysed, 50.3% of the observations were response repetitions and 50.3% were context repetitions.

#### Design

The design was identical to Experiment 1, that is, a 2 × 2 × 2 within-subjects design. To test our hypotheses, we ran two ANOVAs with reaction times or error rates as the dependent variables, and task relation (repetition vs. switch), response relation (repetition vs. switch), and context relation (repetition vs. switch) as the within-subjects independent variables.

As in Experiment 1, we additionally calculated t-tests comparing response repetition and response switch trials in task repetitions in either context repetitions or context switches.

### Results

#### Reaction times

We applied the same data cleaning procedure as for Experiment 1 and this led to removing 13.3% of the observations.

We found a main effect of task relation, *F*(1, 42) = 48.51, *p* < 0.001, η_p_^2^ = 0.536, indicating task-repetition benefits (876 ms vs. 933 ms, for task repetitions vs. switches, respectively). Task relation interacted with response relation, *F*(1, 42) = 13.45, *p* < 0.001, η_p_^2^ = 0.243, so that RR benefits in task repetitions (15 ms) became negative (i.e., costs) in task switches (–17 ms). The main effect of response relation was not significant, *F*(1, 42) = 0.05, *p* = 0.832, η_p_^2^ = 0.001.

The main effect of context relation was significant, *F*(1, 42) = 13.33, *p* < 0.001, η_p_^2^ = 0.241, indicating context repetition benefits (894 ms vs. 915 ms, for context repetition vs. switches, respectively). Context relation entered into significant two-way interactions with task relation, *F*(1, 42) = 9.44, *p* = 0.004, η_p_^2^ = 0.184, and with response relation, *F*(1, 42) = 7.92, *p* = 0.007, η_p_^2^ = 0.159. Both interactions were further qualified by a significant three-way interaction of context relation, task relation, and response relation, *F*(1, 42) = 9.61, *p* = 0.003, η_p_^2^ = 0.186, which we dissected by running separate ANOVAs for context repetitions and context switches. In context repetitions, task relation significantly interacted with response relation, *F*(1, 42) = 18.84, *p* < 0.001, η_p_^2^ = 0.31, so that RR benefits in task repetitions (40 ms) turned into costs in task switches (–18 ms). In context switches, the interaction of task relation and response relation was not significant, *F*(1, 42) = 0.28, *p* = 0.596, η_p_^2^ < 0.01, and we found RR *costs* in both task repetitions (–11 ms) and switches (–16 ms).

We tested the significance of RR effects in task repetitions, both for context repetitions as well as for context switches. The RR benefits were significant in context repetitions (40 ms, *t*(1,42) = 3.98, *p* < 0.001, *d_z_* = 0.61), but not in context switches (-10 ms, *t*(1,42) = –1.26, *p* = 0.214, *d_z_* = 0.19. The Bonferroni-adjusted alpha was equal to .025).

#### Error rates

Applying the same data cleaning as in Experiment 1, we removed 8.3% of the observations. The main effect of task relation was significant, *F*(1, 42) = 51.03, *p* < 0.001, η_p_^2^ = 0.549, indicating task repetition benefits (4.4% vs. 6.75% in task repetitions and switches respectively). Task relation significantly interacted with response relation, *F*(1, 42) = 10.82, *p* = 0.002, η_p_^2^ = 0.205, so that RR benefits in task repetitions (3.6%) were greater than in task switches (1.1%). No other main effect or interaction was significant (all *F*s < 0.84, all *p*s > 0.365).

Although the three-way interaction of task relation, response relation and context relation was not significant, we ran two exploratory paired-sample t-tests on the RR benefits in task repetitions. These were neither significant in context repetitions (1.25%, *t*(1,42) = 1.97, *p* = 0.055, *d_z_* = 0.30), nor in context switches (1.17%, *t*(1,42) = 1.6, *p* = 0.116, *d_z_* = 0.24. The Bonferroni-adjusted alpha was equal to .025).

### Discussion

**Figure 3:**
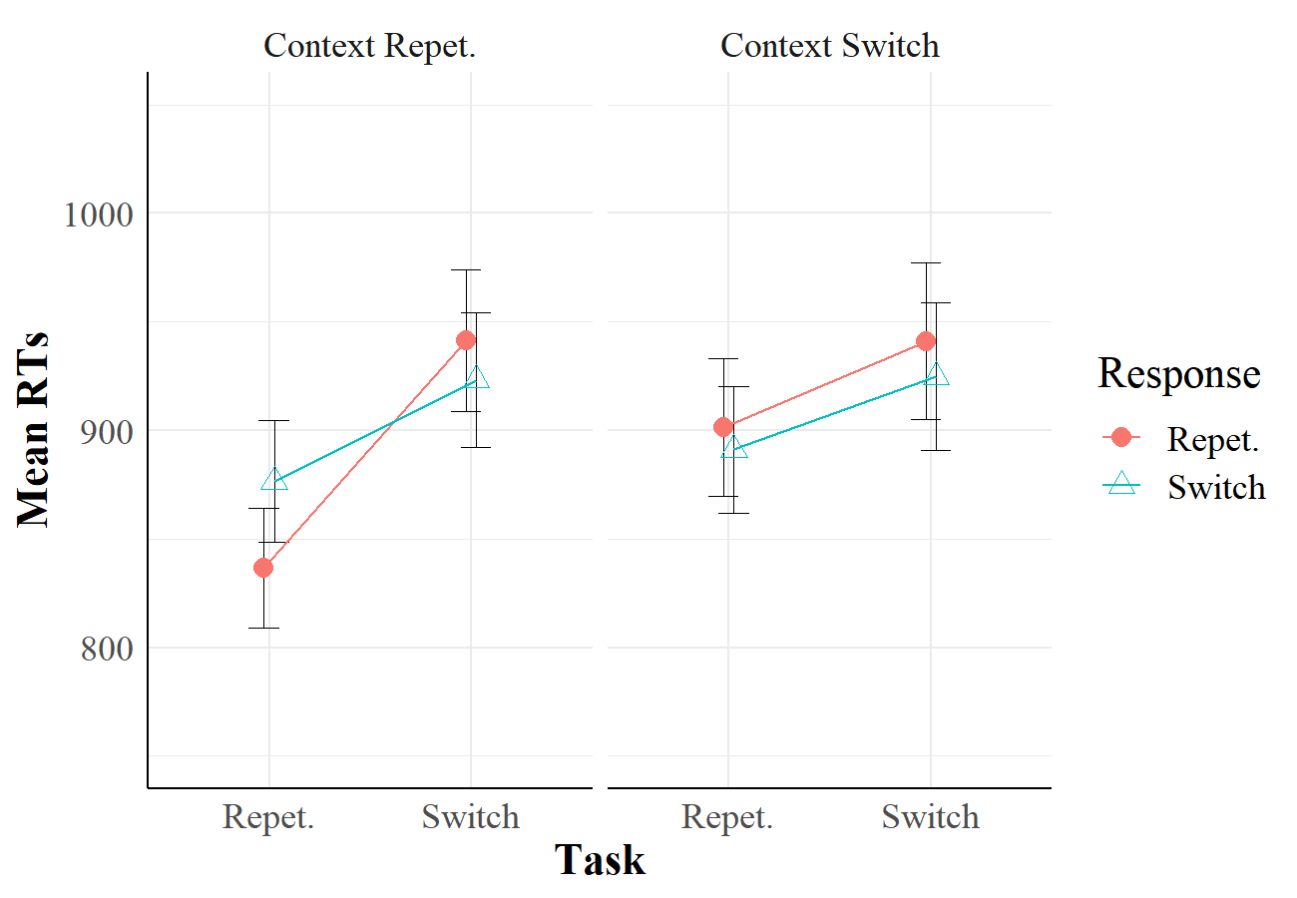
Mean RTs in Experiment 2. *Note*: Vertical bars represent standard errors of the mean.

**Figure 4:**
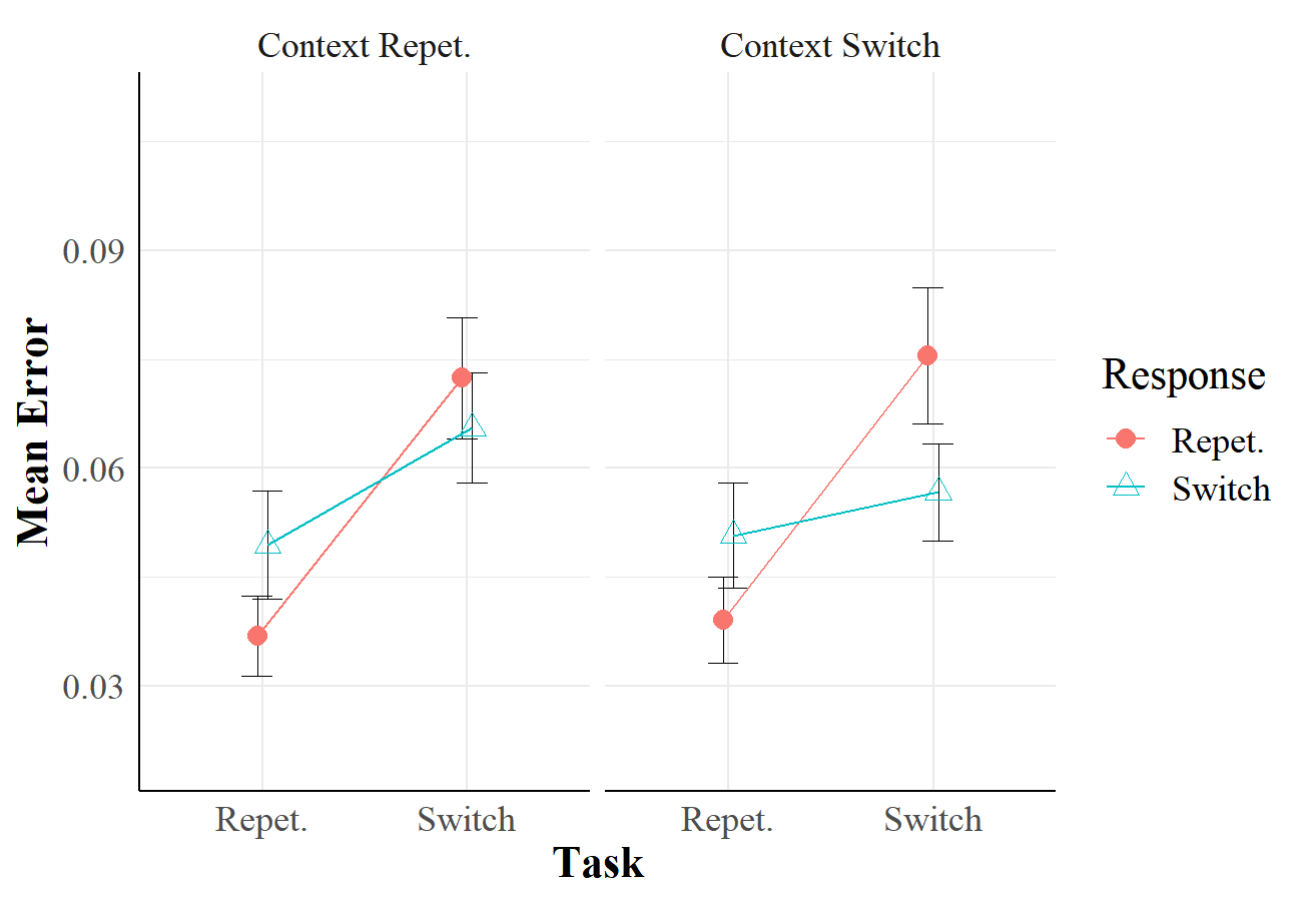
Mean Error Rates in Experiment 2. *Note*: Vertical bars represent standard errors of the mean.

In Experiment 2 (***Figures 3*** and ***4***), we replicated task-repetition benefits and the RR effect as in Experiment 1. Importantly, in the RTs, context relation interacted with task relation and response relation so that the RR benefit in task repetitions was larger when the irrelevant context repeated than when it switched (see ***Figure 3***). This pattern is consistent with the hypothesis that the context is bound with the task and the response in each trial (cf. Koch et al., 2018), and that it can retrieve them when the context repeats in the subsequent trial. Such a retrieval implies a performance benefit when the retrieved features match with the current features.

## General Discussion

In the present study, we investigated whether a task-irrelevant feature (the context), was bound with the task and the response in a task-switching setting. Specifically, along the lines of the TEC (Hommel, 2019) and BRAC (Frings et al., 2020), we examined whether a context that repeats from the previous trial is bound with, and thus retrieves, the previous task and response. Evidence for such a mechanism is the interaction of context relation (repetition vs. switch) with task relation and response relation. If repeating the same response to the same task is especially easier (i.e., faster and/or more accurate) when also the context repeats than when it switches, this is consistent with the context being bound with the task-relevant features and retrieving them when it repeats.

We found statistical support for a repeating context modulating the RR benefits in task repetitions in the RTs in Experiment 2, but not in Experiment 1. However, in the RTs in both experiments, the response-repetition (RR) benefit in task repetitions was significantly greater than zero in context repetitions, but not in context switches.

The pattern of Experiment 2 is coherent with previous studies (cf. Benini et al., 2022; Koch et al., 2018), which also found such a reduction in the RR benefits in task repetitions when the irrelevant context switched compared to when it repeated. Importantly, previous studies used different materials and operationalizations of the context (Benini et al., 2022, used the colour of the cue, and Koch et al., 2018 used cue modality but the cues were shapes vs. tones). Therefore, by finding the same pattern, our study provides additional evidence that a task-irrelevant feature can be bound with, and retrieve the previous-trial task and response in a task-switching setting.

### Binding of irrelevant abstract features

More importantly, to our knowledge, Experiment 2 is the first experiment in which a change in the context did not imply a change at the superficial (perceptual) level of the stimuli. When cue language switched from Spanish to English (or vice versa), participants would always see a black word of similar length (and thanks to surrounding the cue words with Zs, the similarity was even augmented compared to showing the words only). Therefore, we showed that not only a perceptual feature but also a linguistic feature can be bound with the task and the response.

Arguably, these findings are consistent with the Spanish or English language being bound with the task and the response when the cue was in Spanish or English, respectively. Naturally, however, we could not disentangle the binding of a whole language lexicon with the binding of the specific cue word. More studies are needed to examine this question, namely, whether repeating a language, without repeating the specific word, causes the same advantage when also the task and the response repeat.

Using language as the context not only paved the way for new research at the boundary between task and language control but also implied pragmatic improvements. By using the language of the cue, it was easy to vary the context on three levels (Spanish, English, German) so that *within* each trial, the context always switched between the cue (Spanish or English) and the target word (German only). This was the case also in Benini et al. (2022), who used three colours (blue or red for the cue, and black for the target). On the contrary, having three contexts was not possible in Experiment 1 (it would have been possible in a lab setting by using for example tactile stimulation as the cue, but not in an online experiment).

Consequently, the data in Experiment 1 might be noisier than in Experiment 2 because half of the trials contained a context switch within trials (when the cue was auditory) and half that contained a context repetition within trials (when the cue was visual). Moreover, a spoken and a written word are more similar than a high-pitched tone and a bar presented in the upper half of the screen (as used by Koch et al., 2018 and Kandalowski et al., 2020). Hence, the repetition of the cue modality might yield a smaller benefit in our experiment (η_p_^2^ = 0.24) than in theirs (respectively, Experiment 1: η_p_^2^ = 0.79; Experiment 2: η_p_^2^ = 0.78 and Experiment 1: η_p_^2^ = 0.57; Experiment 2: η_p_^2^ = 0.43). Finally, while the cue (hence the context) disappeared just before target presentation in Experiment 1 of the present study, it remained on the screen till the response in Experiment 2. We argue that these reasons contributed to the nonsignificant interactions in Experiment 1.

### The possible impact of feature hierarchy and cue encoding processes

Consistently with Benini et al. (2022), Kandalowski et al. (2020) and Koch et al. (2018), the RR costs in task switches were not affected by a context repetition versus switch. As we discussed in the introduction, this finding is consistent with the co-existence of inhibition of the response (Hübner & Druey, 2006) and binding and retrieval effects in task switching. Furthermore, this finding is also consistent with a hierarchical representation of the features, if one assumes that the task is represented on a hierarchically higher level than the irrelevant context. Hence, a task switch prevents a repeating context from retrieving the previous trial, because a switch of the higher feature implies a reconfiguration of the lower features (see Kleinsorge, 1999), or because it renders the current episode distinct from the previous one (Whitehead et al., 2020).

All in all, our main finding is the increased RR benefits in task repetitions when the context repeated compared to when it switched. An alternative explanation of such a finding is that participants could have adopted a 2:1 cue:task mapping strategy. That is, they could have re-interpreted our instructions considering that each task was signalled by two cues (i.e., one visual and one auditory, in Experiment 1 and one in English and one in Spanish, in Experiment 2). If this was the case, our results reflected cue-repetition benefits in task repetitions but not in task switches. Indeed, performance was the best in cue repetitions (i.e., task and context repetitions), followed by task repetitions (i.e., task repetitions and context switches) and, finally, task switches (i.e., task and context switches).

However, response relation is usually not considered in studies in which the cue can change in task repetitions (Altmann, 2006; Forrest et al., 2014; Logan & Bundesen, 2003; Schneider & Logan, 2011, but see Mayr & Kliegl, 2003) or is examined only indirectly (Schneider & Logan, 2005) or as ancillary analyses (e.g., Arrington & Logan, 2004). Therefore, the present study contributes to filling this gap, by showing a larger impact of cue repetition benefits on response repetition than on response switches. Certainly, further research is needed to disentangle the influence of context relation on task and response relations.

## Conclusion

Taken together the two experiments showed that participants’ performance, mainly in the RTs, was affected by switching versus repeating the task-irrelevant context. Namely, the RR benefit in task repetitions was larger when the context also repeated than when it switched – irrespective of the specific operationalization of the context as cue modality or cue language. This finding is consistent with previous studies examining context binding and retrieval effects in task switching. Our main contribution consisted in extending the generalizability of such findings to different types of context features. Specifically, we demonstrated that a context that varies at a linguistic level but not at a superficial level (words of similar length, same ink colour, but in Spanish vs. English) is bound with, and can retrieve the previous task and response. This finding advocates for the ubiquity of binding and retrieval processes in task switching as suggested by recent theorizing (Frings et al., 2020), which can be observed with different operationalizations of the context.

## Data Accessibility Statement

All the data (*http://dx.doi.org/10.23668/psycharchives.5240*) and the code used to run the analyses (*http://dx.doi.org/10.23668/psycharchives.5239*), including auxiliary analyses, are made available online at the cited DOIs. The study materials are available on request from the corresponding author.

## Footnotes

On a screen with a resolution of 1920 × 1080 and with the browser zoom set to 100%, the words were approximately 6 mm high. The cue words were about 3 cm long, while the target-word length depended on the word and varied between 2.8 and 5.4 cm. The size in which the stimuli were displayed depended on participants’ monitors.

The number of Zs in front (or at the end) of each cue word could be used to recognise the tasks, since two Zs signalled the bigger/smaller task and three Zs the living/non-living task. However, we argue that it is unlikely that participants adopted a “Zs counting strategy” as this requires a perceptual effort bigger than reading the word in the middle on the Zs. Second, participants would abandon the transparent cues that we provided, that is the four words, in favour of rather arbitrary, non-transparent cues, that is the number of Zs. Third, the words were first presented without the Zs in the instructions, which highlighted their semantical link to the tasks at the expense of their visual appearance.

The first 23 participants did not undergo this pre-screening and were included based on their self-reported level of Spanish which must be A2 or higher.

## Author’s note

Some of the data reported in the manuscript were presented at the 10^th^ edition of the A-Dok (Doktorand:innen Workshop, Doctoral Students Workshop) in July 2021.
